# Psychotic symptoms in social anxiety disorder patients: report of three cases

**DOI:** 10.1186/1755-7682-4-12

**Published:** 2011-04-10

**Authors:** André B Veras, Júlia S do-Nascimento, Regis L Rodrigues, Ana Carolina A Guimarães, Antonio E Nardi

**Affiliations:** 1 Panic & Respiration Laboratory, Institute of Psychiatry, Federal University of Rio de Janeiro, INCT Translational Medicine (CNPq), Brazil

## Abstract

**Background:**

Social Anxiety Disorder (SAD) is mainly characterized by an individual's intense concern about other people's opinion of the individual. Notably, among those with severe anxious symptoms, we can often observe self-referential feelings.

**Objective:**

Faced with little research directed toward the exploration of psychotic symptoms in SAD patients, we will approach the topic by describing three cases.

**Discussion:**

Three explanations seem possible for the psychotic manifestations in SAD. The first one depends on the individual's ability or inability to challenge the impression of being criticized by people. A second possibility would be the stressor and perpetuating role of SAD, which would make individuals more likely to present with more severe mental disorders such as delusional disorder (DD). The third explanation would be the possibility that SA is caused by a primary thought abnormality (psychotic self-reference) in some cases, instead of an affective disturbance (anxious insecurity), which led to intense concern about others' opinions. We also observed that antipsychotics did not produce significant improvement in any of the three cases. This result may be related to dopaminergic circuits and the D2 receptor hypoactivity.

**Conclusion:**

The differentiation between delusion and anxious concern may be inaccurate and may change throughout the disorder's evolution. New diagnostic subcategories or the enlargement of the social anxiety diagnostic is proposed to overcome the current diagnostic imprecision. There seems to be a symptomatic spectrum between SAD and DDs.

## Background

Social Anxiety Disorder (SAD) is mainly characterized by an individual's intense concern about other people's opinion of the individual. This concern frequently produces avoidance of and gradual divestment in social relationships. The resulting reclusion produces a common diagnostic difficulty in distinguishing between SAD and schizophrenia spectrum disorders, specifically cluster A personality disorders. To distinguish between these disorders, clinicians usually evaluate the individual's interest in relationships, which is present in SAD, whereas affective indifference is characteristic of schizophreniform disorders. However, although these criteria provide adequate method of distinguishing these disorders in most cases, in some clinical situations, there is interpenetration of these disorders.

So far, many studies have been dedicated to identifying social anxiety (SA) symptoms in schizophrenic patients. Among the most recent studies, given the frequent relationship between these disorders observed in the literature, Lysaker et al. [1] identified that negative symptoms and self-stigma predicted SA symptoms. The same authors [2], in another study, observed that greater paranoia intensity correlated with stronger SA symptoms in schizophrenic patients, as indicated from their better performances of mind theory (the ability to understand others and one self's mental states). In light of these findings, the social relationships and affective importance criteria seem to be weakened. This weakening is due to a wide variability in affective indifference among schizophrenic patients, which is even wider among individuals with cluster A personality disorders or with higher affective preservation, like delusional disorder (DD) patients. This indifference may not even occur, or it can be inversely exacerbated, especially if the patient is aware of his cognitive, affective and social limitations.

Little investigation of psychotic symptoms in SAD patients has been reported in the literature. Notably, among those with severe anxious symptoms, we can often observe self-referential feelings. Such feelings can be accompanied by more or less criticism by the patient of the symptom. The less insight the individual has that this feeling is due to the patient's thoughts, the closer to delusion this symptom gets. As Martin and Penn [3] have shown, in a nonclinical sample, greater paranoid ideation is associated with higher levels of social anxiety, avoidance, apprehension about evaluation, self-observation and low self-esteem. An interesting relationship between anxiety and psychosis was observed by Freeman and Fowler [4]. These authors found that a trauma history report influences the intensity of persecutory thoughts through anxiety generation and that anxiety is also responsible for the relationship between substance use and paranoia.

Faced with little research directed toward the exploration of psychotic symptoms in SAD patients, we will approach the topic by describing three cases. These cases were outpatients at an anxiety clinic linked to the National Institute for Translational Medicine, Brazil [5].

## Cases Presentation

*Case 1 *was a 30-year-old male, seeking treatment for secondary use of smoked cocaine (crack). He had moderate depressive symptoms and significant anxiety with physical manifestations. His longitudinal history report included intense nervousness, accompanied by palm sweating, tachycardia and face flushing if exposed to simple social interactions (e.g., requesting information on the street or at work). Such events generated avoidance behavior and produced failure in love relationships. Crack use helped relieve those discomforts. These clinical features led to a social phobia diagnosis, favoring dependence on a cocaine derivative and a current depressive episode. We started the patient on fluoxetine (20 mg/day) and clonazepam (1.5 mg/day) and recommended that he attend a support group and individual psychotherapy. He remained abstinent for 60 days but had a slight relapse afterward. During a second period of abstinence, after about 45 days, he became increasingly concerned about his neighbors' opinion. He believed that local youths, with whom he had previously been involved in a physical fight in the context of narcotics consumption, mocked and teased him with the intention of fighting again. He then became involved in a fight in a commercial establishment near his house after he felt provoked. He mentioned that one of the individuals who "provoked" him entered the store and asked aloud if the seller sold cigarettes. The patient concluded that the question could only be a provocation aimed at him, because smoking was not allowed at the site. Thus, before he defiantly "lit a cigarette to cope with him", the patient started a discussion that culminated in a physical struggle. Soon after the event, the patient relapsed on crack use. He sought psychiatric treatment again, and only after the elucidation of his symptoms did he express criticism of the self-referential feeling and the delusional interpretation.

*Case 2 *was a 42-year-old male of Eastern descent. The patient sought treatment for severe anxiety related to going out alone on the street. In these situations, he had the feeling that people would comment about him and mock him. He also had an inability to maintain interpersonal relationships due to anxiety with symptoms such as palpitations, tremor and sweating. The comments and jeers that he feared referred to questions about his sexual orientation. He also had dystonic neck movement, which started after the temporary use of haloperidol during a previous treatment and which worsened his social anxiety. He was maintained using cloxazolam (6 mg/day), which he was already taking, and was started on fluoxetine (up to 40 mg/day). Despite the reported reduction in psychological anxiety, the patient did not improve his ability to establish social relationships or reduce his concern about receiving negative comments about his sexuality. In fact, these concerns intensified, and he started to have a strong impression that the neighbors and passers-by laughed when he passed and made comments that indirectly questioned his sexual orientation. Such feelings occurred only when he walked alone on the street. He was convinced that he was being harassed by the neighborhood, and he felt very threatened. He started to interpret noises produced in the vicinity of the house as provocations of him and his father, with whom he lived. Faced with such intense discomfort, he reported that it required great courage to leave home and "face the world". On one occasion, when entering a supermarket, he was certain that he had been cursed by an unknown person who sneezed by his side, interpreting the sound of the sneeze as the word "queer." Given these facts and the persistence of tardive dystonia, he was started on quetiapine (50 mg/day), scheduled for gradual dose adjustment according to tolerability. Even with all the psychotic manifestations described, the patient maintained his usual level of function.

*Case 3 *was a 39-year-old female. She was referred to psychiatric attention because of three months of depressive symptoms, such as sadness and a lack of a will to live. These symptoms were motivated by the belief, which she had held for six months, that people loathed and kept away from her because she exhaled a fetid smell. She had the impression that people avoided or laughed at her when she walked on the street. Because she could not find a reason for such conduct, she tried to convince herself that it was a false impression. Nevertheless, she began to worry about her smell until, during a family meeting, one of the cousins said the patient was "stinking" and should stay away from him on the picture. Since then, the patient believed that she had a foul odor that, although she herself did not smell it, was noticeable to others. Then, she started to wake up very early to catch the first available public transportation, avoiding further contact with people. When questioned about the existence of anxiety symptoms throughout life, she said she never talked to a classmate at school and only four years ago could eat in the presence of strangers, so she rarely ate during a workday due to shame. The only intimate physical contact she allowed was hugging her mother and sisters on their birthdays and Christmas. Due to loss of appetite and intense insomnia, she was started on amitriptyline (up to 125 mg/day) and diazepam (5 mg/day). The patient presented partial remission of the anxiety symptoms and mood depression. She also used risperidone (up to 2 mg/day), which did not result in improvement and worsened the conviction that she smelled bad. The antipsychotic was switched to haloperidol (3 mg/day). Then, her delusional ideation improved. She showed extrapiramidal symptoms and weight gain. The antidepressant was switched to fluoxetine (up to 60 mg/day). Gabapentine (900 mg/day) was added for therapeutic potentiating. The patient's family reported that gabapentine was the drug that most reduced the severity of the avoidant behavior at home. In recent appointments, she has again expressed the thought that everyone avoids her and the conviction that the only possible reason is her smell.

## Discussion

Three explanations seem possible for the psychotic manifestations in SAD. The first one depends on the individual's ability or inability to challenge the impression of being criticized by people. This ability can lead to two poles of symptoms:

1. The greater an individual's ability to recognize that the idea is exaggerated, the more the idea resembles an anxious concern like an obsession. That finding is consistent with a symptomatic presentation described in the literature, which have similarities with the third case. The olfactory reference syndrome [6] is characterized by delusions that the individual exude a foul odor, although it does not actually occur. The carriers of the disorder tend to imagine that your breath, underarms and genitals are with an unbearable stench for people lining with them, taking them to isolation due to this belief. Due to these delusions they also take several showers a day and wash their clothes they wear obsessively. Hence this disorder also be considered as part of the spectrum of obsessive compulsive disorder.

2. The less insight, the greater the belief that the idea is a reality, the more the experience resembles a delusional self-reference. This delusion was present in the first case in which the patient gradually developed a conviction that he was harassed by the neighbors while he repeatedly exposed himself to situations that reinforced this feeling. Later, when he challenged the delusional idea and obtained some insight, he became able to consider the absurdity of his thinking. The role of use of derivatives of cocaine for the occurrence of psychotic symptoms in the first case can not be neglected. However, this element seems to be at best a partial adjuvant. This can be affirmed because the patient had symptoms while standing up for some months abstinent. The relapse occurred because of the psychological discomforts described. Although it is possible the presentation of psychosis later, the most common manifestation is related to concomitant drug use or during the withdrawal phase [7].

A second possibility would be the stressor and perpetuating role of SAD, which would make individuals more likely to present with more severe mental disorders such as DD. This occurrence seems consistent with the third case. The case's evolution with delusion maintenance and poor response to antipsychotic treatment may be considered to be usual paranoia. It seems as though the second case also evolved with the occurrence of DD, although its evolution and response to treatment has not been observed. Michail and Birchwood [8] have also raised this hypothesis by observing that psychotic patients with SAD had more vivid feelings that "someone had the aim to harm" them (45% vs. 11.6%) compared with psychotic patients without SAD.

The third explanation would be the possibility that SA is caused by a primary thought abnormality (psychotic self-reference) in some cases, instead of an affective disturbance (anxious insecurity), which led to intense concern about others' opinions. Faced with subsyndromal manifestations, the clinical differentiation among these conditions would be particularly difficult. Because of the self-reference, interpersonal relationships would cause particular concern and discomfort, thus causing the individual to develop an avoidant personality. Despite not belonging to cluster A, we suggest that the avoidant personality of cases 2 and 3 could also be seen as a pre-morbid functioning paranoia. This possibility is consistent with that raised by Michail and Bischwood [8] that "social anxiety and persecutory thinking develop concurrently in the early phase of psychosis and follow a similar course".

The difficulty in distinguishing SA from psychosis in some cases reveals the fragility of diagnostic constructs and the current psychopathological models. Of the third case's particular situation, some authors [9] have observed this presentation as an "offensive SAD subtype", which also includes patients with (delusional) conviction of offensiveness. Such concepts are the product of a syndrome recognized in Japan since 1930 as "Taijin-Kyofu" (TK) [10]. Its "conviction subtype" is characterized by a strong belief and fear that others are disturbed by the individual's inadequacy. This inadequacy could occur in several ways due to the emission of body odors, facial expressions, intestinal noises, and so forth [9]. Among Western psychiatrists guided by the DSM-IV, such a condition can only be diagnosed as a DD. Still, TK has been described in some Anglo-Saxon countries, such as Australia [11] and the U.S. [12]. In view of this, we suggest that the "conviction subtype TK" may represent a categorical model for patients placed between SAD and the DDs at a spectral view.

We observed that antipsychotics did not produce significant improvement in any of the three cases. This result may be related to dopaminergic circuits and the D2 receptor hypoactivity observed in SAD patients [13,14]. Therefore, from the neuro-pathophysiologic standpoint, it would make little sense to reduce the function of a system already in deficit by blocking dopamine's action with an antipsychotic. It is important to note that in the second case, typical antipsychotics worsened the outcome due to the occurrence of tardive dystonia. A series of case reports corroborate the lower efficacy of using antipsychotics in SAD, even with the occurrence of delusions. They observed better effectiveness of selective serotonin and serotonin and norepinefrine reuptake inhibitors on conviction-subtype TK patients [12,15-18]. On the other hand, in the third case reported, only risperidone was used as atypical agent. Thus, despite the poor response to antipsychotics prescribed, the condition can not be considered refractory, once the prescription of other atypical antipsychotic could generate satisfactory therapeutic response.

## Conclusion

In this article, we observe the occurrence of psychotic symptoms in patients with SAD. The differentiation between delusion and anxious concern may be inaccurate and may change throughout the disorder's evolution, as observed in the three cases described. Moreover, the response to antipsychotics proved to be poor. The addition of new diagnostic subcategories or the enlargement of the social anxiety diagnostic is proposed to overcome the current diagnostic imprecision. There seems to be a symptomatic spectrum between SAD and DDs.

## Consent

Written informed consent was obtained from the patient for publication of this case report and any accompanying images. A copy of the written consent is available for review by the Editor-in-Chief of this journal.

## Competing interests

The authors declare that they have no competing interests.

## Authors' contributions

ABV, JSN, RLR and ACAG: acquisition of data, translation and analysis and interpretation of data; AEN: acquisition of data, translation and analysis, interpretation of data and general supervision. All authors read and approved the final manuscript.
